# Role of the Transcranial Doppler Bubble Test in the Diagnostic Pathway of a Patent Foramen Ovale in Embolic Stroke of Undetermined Source

**DOI:** 10.7759/cureus.104051

**Published:** 2026-02-22

**Authors:** Hajar Khattab, Hajar El Omari, Manal Khalfi, Asmae Sikkal, Kamal Haddouali, Salma Bellakhdar, Hicham El Otmani, Bouchra El Moutawakil, Mohammed Abdoh Rafai

**Affiliations:** 1 Department of Neurology, Ibn Rochd University Hospital - Hassan II University, Casablanca, MAR; 2 Department of Neurology, Ibn Rochd University Hospital – Hassan II University, Casablanca, MAR

**Keywords:** patent foramen ovale, secondary stroke prevention, stroke, transcranial doppler, transoesophageal echocardiography

## Abstract

Background: A patent foramen ovale (PFO) is frequently found in embolic stroke of undetermined source (ESUS). The transcranial Doppler (TCD) bubble test allows for the sensitive detection of right-to-left shunt but is still underused in standard diagnostic procedures.

Objective: This study aimed to evaluate the clinical impact of the TCD bubble test on diagnostic reclassification and secondary prevention strategies in patients with ESUS.

Methods: We conducted a retrospective observational study including ESUS patients admitted to the Department of Neurology, Ibn Rochd University Hospital, a tertiary care neurology center in Casablanca, Morocco, between January 2023 and July 2025. All patients underwent the TCD bubble test. Transesophageal echocardiography (TEE) was performed when clinically indicated. Right-to-left shunt severity was graded according to international consensus criteria. PFO was considered confirmed only when functional evidence on TCD and anatomical confirmation on TEE were concordant. Clinical, imaging, and therapeutic data were analyzed descriptively.

Results: A total of 117 patients met the inclusion criteria (mean age 45.5 ± 12.4 years; 73/117 (62.0%) female). TCD detected a right-to-left shunt in 48/117 patients (41.0%), with a severe shunt or curtain effect observed in 23/117 patients (19.7%). An adequate Valsalva maneuver was achieved in 109/117 examinations (93.2%). TEE was performed in 59/117 patients (50.4%). Initial discordance between TCD and TEE was observed in 13/59 patients (22.0%). Repeat TEE, performed in selected discordant cases, led to diagnostic correction in all re-evaluated patients, increasing final concordance to 54/59 (91.5%). Overall, PFO was confirmed in 39/117 patients (33.3%). In one patient with a severe shunt on TCD and a negative TEE, further evaluation revealed a pulmonary arteriovenous malformation. TCD findings directly influenced clinical management, including antithrombotic strategy selection and referral for percutaneous PFO closure. Percutaneous closure was performed in 22/117 patients (18.8%), including selected individuals aged ≥60 years with high-risk functional and anatomical features.

Conclusions: In patients with ESUS, the TCD bubble test provides valuable clinical information that corrects diagnostic errors and directly influences secondary prevention strategies. By guiding the use and interpretation of TEE and supporting personalized treatment decisions, TCD acts as an effective first-line screening and gatekeeper tool in ESUS diagnostic pathways.

## Introduction

A patent foramen ovale (PFO) is a fetal connection between the atria that typically closes after birth; an anatomical PFO persists in about 25% of adults [1]. PFOs are more frequently found in patients with cryptogenic ischemic stroke compared to the general population, especially among younger adults [2].

The concept of embolic stroke of undetermined source (ESUS) was developed to identify an essential subgroup of cryptogenic stroke believed to be caused by thromboembolism [3]. ESUS requires a non-lacunar infarct on neuroimaging, absence of ≥50% narrowing in arteries supplying the affected area, no primary cardioembolic source (e.g., atrial fibrillation, intracardiac thrombus, prosthetic valve), and the completion of a minimum diagnostic work-up (brain CT/MRI; 12-lead ECG; transthoracic echocardiography; extracranial and intracranial vascular imaging; and, when necessary, ambulatory rhythm monitoring and routine laboratory tests). This practical definition highlights causes with embolic potential and impacts treatment decisions.

Among ESUS cohorts, PFO prevalence is higher than in the general population. It varies with age and detection method, generally ranging from about 25-50%, with the highest estimates in younger patients [4]. In addition, the likelihood of a causal relationship depends on shunt size and high-risk anatomical features, such as atrial septal aneurysm, which also inform secondary prevention strategies, including considering percutaneous closure in selected cases [4].

Transesophageal echocardiography (TEE) with agitated-saline contrast remains the standard test for assessing PFO anatomy and visualizing right-to-left microbubble transit, but its invasiveness, sedation needs, and limited effectiveness with suboptimal Valsalva can lower sensitivity [5]. By contrast, the transcranial Doppler (TCD) bubble test is non-invasive, easy to perform at the bedside, and highly sensitive for detecting right-to-left shunt, with good tolerability; however, it does not provide anatomical localization of the shunt [6].

This study aimed to evaluate the diagnostic value of the TCD bubble test as a screening method for PFO in patients with ESUS and its influence on clinical decisions, such as referring for TEE and considering percutaneous closure, as well as its effect on treatment timelines.

## Materials and methods

Study design and population

We conducted a retrospective observational study at the Department of Neurology, Ibn Rochd University Hospital, Casablanca, including patients admitted between January 2023 and July 2025. All patients aged ≥16 years admitted for acute ischemic stroke during the study period were screened.

Among 1,200 consecutive patients hospitalized for ischemic stroke, those with a major determined etiology or not meeting criteria for ESUS were excluded. After this initial selection, 300 patients fulfilled the ESUS definition. Patients without a TCD examination with a bubble test, or with missing or incomplete data, were subsequently excluded, resulting in a final study population of 117 patients.

Definition of ESUS

ESUS was defined according to the criteria outlined by Hart et al. (2014) [7] as (1) a non-lacunar ischemic infarct on CT/MRI; (2) absence of ≥50% stenosis in arteries supplying the ischemic territory; (3) no major-risk cardioembolic source (e.g., atrial fibrillation, intracardiac thrombus, mechanical valve); and (4) completion of a minimum diagnostic work-up including brain imaging, 12-lead ECG, transthoracic echocardiography, and intracranial/extracranial vascular imaging, with rhythm monitoring as clinically indicated.

All patients underwent a standardized diagnostic assessment that included a 12-lead electrocardiogram, 24-hour Holter ECG monitoring, transthoracic echocardiography, and computed tomography angiography of the supra-aortic trunks. Laboratory tests included a complete blood count, erythrocyte sedimentation rate, C-reactive protein, basic metabolic panel, viral serologies, and minimal autoimmune screening (antinuclear antibodies and anti-double-stranded DNA antibodies). Patients with atrial fibrillation, significant arterial stenosis, or other major causes of ischemic stroke were excluded.

Neuroimaging assessment

All patients underwent brain magnetic resonance imaging (MRI) using a standardized vascular protocol that included diffusion-weighted imaging (DWI), fluid-attenuated inversion recovery (FLAIR), T2*-weighted sequences, and time-of-flight magnetic resonance angiography (TOF-MRA). An experienced neuroradiologist and a vascular neurologist independently reviewed MRI scans.

TCD bubble test

Cervical and transcranial examinations were carried out by a certified neurosonologist using the same ultrasound system (Philips Affiniti 70). Transcranial insonation was performed with a 3-5 MHz probe through the right and left transtemporal windows, and through the occipital window when needed.

Agitated saline contrast was injected both at rest and during a standardized Valsalva maneuver. Adequate Valsalva was defined as a ≥30% decrease in mean systolic velocity in the middle cerebral artery. Right-to-left shunt was identified by the appearance of high-intensity transient signals (HITS) after contrast injection and graded according to International Consensus recommendations for contrast-TCD shunt detection and microbubble count categorization [8]: grade 0 (no HITS), grade I (1-10 microembolic signals), grade II (11-30 signals), grade III (>30 signals without curtain), and grade IV (curtain or shower pattern). The highest grade observed at rest or during Valsalva was used for analysis.

Transoesophageal echocardiography

TEE was performed when clinically indicated, primarily in patients with a positive TCD or in cases of inconclusive TCD results (such as poor acoustic window, ineffective Valsalva maneuver, or technical limitations). TEE aimed to characterize PFO anatomy, including shunt size and the presence of an atrial septal aneurysm, and to identify alternative cardioembolic sources. During contrast TEE, an intracardiac shunt was suggested by the appearance of microbubbles in the left atrium within the first three cardiac cycles after right atrial opacification, whereas delayed appearance raised suspicion of an extracardiac shunt.

TEE reports systematically included assessment of cardiac chamber dimensions, ventricular contractility, and left ventricular ejection fraction, as well as valvular abnormalities, intracardiac thrombi or vegetations, pulmonary venous drainage, ascending aorta, and pericardium. Experienced cardiologists conducted all examinations. In cases of discordance between TCD and TEE findings, a repeat TEE performed by a different cardiologist was encouraged.

Management of discordant TCD-TEE findings

In cases of discordance between contrast-enhanced TCD and transesophageal echocardiography, a predefined stepwise diagnostic approach was applied.

When TCD was positive and the initial TEE was negative, the decision to perform a repeat TEE depended on the severity of the shunt detected on TCD. In patients with a moderate or large right-to-left shunt, a second TEE was systematically performed by a different cardiologist, with particular attention to contrast injection technique and optimization of the Valsalva maneuver.

If the repeat TEE remained negative despite a moderate or large shunt on TCD, patients underwent thoracic computed tomography angiography to investigate alternative extracardiac sources of right-to-left shunt, particularly pulmonary arteriovenous malformations.

In patients with a minimal shunt on TCD and persistently negative TEE findings, the presence of a small PFO not detectable by transesophageal echocardiography was considered the most likely explanation.

PFO definition and risk stratification

For this study, a PFO was considered confirmed only if both functional evidence of a right-to-left shunt on the TCD bubble test and anatomical confirmation on transesophageal echocardiography were present. High-risk anatomical features of PFO included a defect larger than 2 mm, an atrial septal aneurysm with septal excursion over 10 mm, the presence of a Eustachian valve, or a large shunt on contrast TEE indicated by passage of more than 30 microbubbles. Functional high-risk criteria involved a severe right-to-left shunt or a curtain effect observed on TCD.

The Risk of Paradoxical Embolism (RoPE) score (0-10) was calculated for each patient using the original RoPE model (points assigned for younger age, absence of vascular risk factors, and cortical infarct pattern) [9]. Higher RoPE scores indicate a higher probability that the PFO is pathogenic rather than incidental.

The PFO-Associated Stroke Causal Likelihood (PASCAL) classification was determined by combining the RoPE score with high-risk PFO features (large shunt and/or atrial septal aneurysm) to categorize the likelihood of a causal PFO-stroke relationship as "probable," "possible," or "unlikely" [10].

Therapeutic management

All patients meeting ESUS criteria received standard medical treatment with single antiplatelet therapy. Patients with high-risk PFO features were treated with anticoagulation while awaiting percutaneous PFO closure. Therapeutic decisions were made during multidisciplinary case discussions involving vascular neurologists, cardiologists, and interventional cardiologists.

Outcome assessment

Functional outcome was assessed using the modified Rankin Scale (mRS) at three and six months during outpatient follow-up visits. Scores range from 0 (no symptoms) to 6 (death); a favorable outcome was defined as mRS 0-2 [11].

Stroke recurrence was defined as the occurrence of a new transient ischemic attack or ischemic stroke confirmed by neuroimaging.

Statistical analysis

Statistical analyses were conducted using R software (R Foundation for Statistical Computing, Vienna, Austria). Quantitative variables are expressed as mean ± SD or median (IQR), as appropriate. The analyses were descriptive, and no formal statistical hypothesis tests or significance levels were used because of the small sample sizes in subgroup comparisons.

Ethics statement

Given the retrospective nature of the study, formal approval from an institutional ethics committee was not required. Oral informed consent was obtained from all patients in accordance with local regulations.

## Results

A total of 117 patients who met the ESUS criteria and underwent the TCD bubble test were included in the final analysis. The average age was 45.5 ± 12.4 years, with a female predominance (62%). Traditional vascular risk factors were rare. A previous ischemic stroke or transient ischemic attack was reported in 15% of patients. A history of migraine was reported in 14 patients (12%). The baseline characteristics of the ESUS cohort are detailed in Table 1.

**Table 1:** Baseline characteristics of the ESUS cohort (n = 117). ESUS: embolic stroke of unknown source, TIA: transient ischemic attack

Variables	n (%)
Age, years, mean ± SD	45.5 ± 12.4
Female sex, n (%)	73 (62%)
Hypertension, n (%)	31 (26%)
Diabetes mellitus, n (%)	20 (17%)
Dyslipidemia, n (%)	12 (10%)
Smoking, n (%)	30 (26%)
Alcohol consumption, n (%)	14 (12%)
Cannabis use, n (%)	14 (12%)
Oral contraception, n (%)	15 (13%)
Previous ischemic stroke or TIA, n (%)	18 (15%)
Venous thromboembolism history, n (%)	1 (0.9%)
Prolonged immobilization or long-distance travel, n (%)	6 (5.1%)
History of migraine, n (%)	14 (12%)

At admission, the median National Institutes of Health Stroke Scale (NIHSS) score was 4 (2-9.25). Loss of consciousness was seen in seven patients (6.0%). Three patients (2.6%) required decompressive craniectomy or cerebrospinal fluid diversion. Clinical characteristics at presentation are detailed in Table 2.

**Table 2:** Clinical characteristics at presentation (n = 117). NIHSS: National Institutes of Health Stroke Scale

Clinical characteristics at admission	n (%)
NIHSS at admission, median (IQR)	4 (2–9.25)
Loss of consciousness, n (%)	7 (6.0%)
Decompressive craniectomy or CSF diversion, n (%)	3 (2.6%)

Brain MRI showed a predominance of carotid territory involvement (61.5%), mainly within the middle cerebral artery region (59.0%). Vertebrobasilar circulation was affected in 42.7% of patients. Multifocal infarctions were seen in 29.1% of cases. Hemorrhagic transformation occurred in 9.4% of patients, and imaging features consistent with ischemic infarcts of different ages were observed in 21.4%. MRI patterns are summarized in Tables 3-4.

**Table 3:** Global MRI characteristics and lesion patterns (n = 117). MRI: magnetic resonance imaging

MRI characteristics	n (%)
Carotid territory involvement	72 (61.5%)
Vertebrobasilar territory involvement	50 (42.7%)
Multifocal infarction	34 (29.1%)
Hemorrhagic transformation	11 (9.4%)
Infarcts of different ages	25 (21.4%)

**Table 4:** Distribution of ischemic lesions according to arterial territories (n = 117).

Arterial territory	n (%)
Middle cerebral artery (MCA)	69 (59.0%)
Anterior cerebral artery (ACA)	8 (6.8%)
Anterior choroidal artery	0 (0%)
Posterior cerebral artery (PCA)	25 (21.4%)
Artery of Percheron	6 (5.1%)
Posterior inferior cerebellar artery (PICA)	21 (17.9%)
Anterior inferior cerebellar artery (AICA)	6 (5.1%)
Superior cerebellar artery (SCA)	5 (4.3%)
Basilar artery perforators	4 (3.4%)
Vertebral artery	3 (2.6%)

The TCD bubble test was performed on all patients. A right-to-left shunt was identified in 48 patients (41.0%). An adequate Valsalva maneuver was achieved in 93.2% of tests. The severity of the shunt ranged from minimal to “curtain effect" with spontaneous “curtain effect” seen in 7.7% of patients. The TCD findings are reported in Table 5.

**Table 5:** Transcranial Doppler (TCD) findings (n = 117).

TCD findings	n (%)
TCD performed	117 (100%)
Positive right-to-left shunt	48 (41.0%)
Good Valsalva cooperation	109 (93.2%)
Valsalva-induced shunt sensitization	32 (27.4%)
Minimal shunt	12 (10.3%)
Moderate shunt	16 (13.7%)
Severe shunt	6 (5.1%)
Curtain effect	14 (12.0%)
Spontaneous curtain effect	9 (7.7%)

TEE was performed in 59 patients (50.4%), with adequate Valsalva maneuver achieved in 76.3%. A PFO was detected on the initial TEE in 35 patients (59.3%). Eight patients underwent a second TEE, which was positive in six cases (75.0%). Overall, PFO was confirmed on the final TEE assessment in 39 patients (66.1%). The mean PFO size was 5.5 ± 3.8 mm, and an atrial septal aneurysm was present in 23.7% of patients. Two patients with initially positive TEE and negative TCD had negative repeat TEEs, leading to final reclassification. The TEE results are shown in Table 6. 

**Table 6:** Transoesophageal echocardiography findings (n=59) Repeat TEE led to reclassification in some patients; therefore, the number of final PFO confirmations does not equal the sum of initial and repeat positive examinations.

TEE findings	Value
TEE performed, n (%)	59 (50.4%)
Good Valsalva cooperation, n (%)	45 (76.3%)
PFO detected on initial TEE, n (%)	35 (59.3%)
Repeat TEE performed, n (%)	8 (13.6%)
Positive repeat TEE, n (%)	6 (75.0%)
PFO confirmed on final TEE, n (%)	39 (66.1%)
PFO size, mean ± SD (mm)	5.5 ± 3.8
Atrial septal aneurysm, n (%)	14 (23.7%)

Among the 59 patients who underwent both TCD and TEE, initial TCD-TEE discordance was observed in 22.0% (13/59). Repeated TEE, performed on eight discordant cases, resulted in diagnostic correction in all patients: six were reclassified as having a previously missed PFO, and two were reclassified as having false-positive initial TEE findings. Final concordance increased from 78.0% to 91.5%. Discordant cases mainly involved low-grade shunts on TCD. Concordance analyses are shown in Tables 7-8.

**Table 7:** Concordance between TCD bubble test and TEE for PFO detection (initial vs. final TEE), among patients who underwent both tests (n = 59). TCD: transcranial Doppler, TEE: transesophageal echocardiography, PFO: patent foramen ovale

Comparison	Concordant n (%)	Discordant n (%)
TCD vs. initial TEE	46 (78.0%)	13 (22.0%)
TCD vs. second TEE	54 (91.5%)	5 (8.5%)

**Table 8:** Reclassification of PFO diagnosis after the second TEE in discordant cases. PFO: patent foramen ovale, TEE: transesophageal echocardiography

Category	n
Initially discordant cases	13
Repeat TEE performed	8
Concordant after repeat TEE	8
• Reclassified as concordant positive	6
• Reclassified as concordant negative	2
Persistent discordance after repeat TEE	0
Discordance without repeat TEE	5

A second TEE was performed as outlined in the study protocol in cases of discordant TCD and TEE findings.

Among the five patients with discordant findings (TCD-positive and initial TEE-negative) who did not undergo repeat TEE, four presented a minimal right-to-left shunt on TCD. At the same time, one had a severe shunt, and none exhibited a curtain effect. Notably, the single patient presenting a severe right-to-left shunt on TCD despite a negative initial TEE was subsequently found to have a pulmonary arteriovenous malformation consistent with hereditary hemorrhagic telangiectasia on chest computed tomography.

Among the 117 patients with embolic stroke of undetermined source included in the study, PFO was ultimately confirmed in 39 patients, which is a prevalence of 33.3% in this ESUS group. PFO confirmation was based on consistent findings from the TCD bubble test and TEE.

Among patients with confirmed PFO, the median RoPE score was 7 (6-9), with 69.2% having a RoPE score of ≥7. The distribution of RoPE scores is presented in Table 9. According to the PASCAL classification, nearly half of the patients were classified as having a probable PFO-related stroke. The PASCAL classification is summarized in Table 10.

**Table 9:** RoPE score distribution in the patients with confirmed PFO (n = 39). RoPE: Risk of Paradoxical Embolism, PFO: patent foramen ovale

Variable	Value
RoPE score, median (IQR)	7 (6–9)
RoPE score ≥7, n (%)	27 (69.2%)

**Table 10:** PASCAL classification of the likelihood of PFO-related stroke among patients with confirmed PFO (n = 39). PASCAL: PFO-Associated Stroke Causal Likelihood, PFO: patent foramen ovale

PASCAL category	n (%)
Probable	19 (48.7%)
Possible	17 (43.6%)
Unlikely	3 (7.7%)

All patients received medical therapy in accordance with ESUS guidelines. Antiplatelet therapy was administered in 69.2% of patients, while 30.8% received anticoagulation pending further evaluation or percutaneous closure. Percutaneous PFO closure was performed in 18.8% of patients. Among the 22 patients who underwent percutaneous PFO closure, the majority were aged 25-59 years (81.8%). Notably, three patients (13.6%) were aged 60 years or older at the time of closure. Therapeutic management strategies, including antithrombotic treatment and percutaneous PFO closure, are summarized in Table 11.

**Table 11:** Therapeutic management. PFO: patent foramen ovale, TEE: transesophageal echocardiography

Management strategy	n (%)
Antiplatelet therapy	81 (69.2%)
Anticoagulation pending closure	36 (30.8%)
PFO closure planned	11 (9.4%)
PFO closure performed	22 (18.8%)
Awaiting TEE for closure decision	3 (2.6%)

Functional outcome was assessed in 86 patients with available follow-up data. At three months, the median mRS score was 1 (0-2), with 77.9% achieving a favorable outcome. At six months, the median mRS score was 0 (0-2), and 73.3% of the patients had a favorable functional outcome. Stroke recurrence occurred in 1.7% of patients during follow-up. Functional outcomes are summarized in Table 12.

**Table 12:** Functional outcomes at follow-up. mRS: modified Rankin Scale, IQR: interquartile range

Outcome	Value
mRS at three months, median (IQR) (n = 86)	1 (0–2)
Favorable outcome (mRS 0–2) at three months	67/86 (77.9%)
mRS at six months, median (IQR) (n = 86)	0 (0–2)
Favorable outcome (mRS 0–2) at six months	63/86 (73.3%)

An exploratory comparison was performed between ESUS patients with a confirmed PFO (n = 39) and ESUS patients without a PFO. The ESUS non-PFO group included only patients with definitive exclusion of PFO based on concordant negative TCD and TEE findings (n = 15); patients without TEE were not included in this subgroup analysis to avoid diagnostic misclassification.

Baseline clinical characteristics are compared between ESUS patients with confirmed PFO and those without PFO in Table 13. Patients with confirmed PFO were less frequently female and had a lower median NIHSS score at admission. A history of migraine was more commonly reported in the ESUS-PFO group, whereas oral contraceptive use appeared more common among ESUS patients without PFO. Prior ischemic stroke or transient ischemic attack was observed in both groups.

**Table 13:** Clinical characteristics of ESUS–PFO versus ESUS non-PFO. ESUS: embolic stroke of unknown source, PFO: patent foramen ovale

Clinical characteristics	ESUS–PFO (n = 39)	ESUS non-PFO (n = 15)
Female sex, n (%)	20 (51.3%)	12 (80.0%)
Migraine, n (%)	6 (15.4%)	1 (6.7%)
Smoking, n (%)	13 (33.3%)	4 (26.7%)
Oral contraception, n (%)	4 (10.3%)	4 (26.7%)
Previous ischemic stroke or TIA, n (%)	6 (15.4%)	3 (20.0%)
NIHSS at admission, median (IQR)	3 (0–5)	5 (2–11)

Regarding neuroimaging findings, brain MRI patterns were broadly comparable between groups. The proportions of multifocal infarction, carotid and vertebrobasilar territory involvement, middle cerebral artery and posterior cerebral artery infarctions, as well as infarctions in the artery of Percheron territory, were similar. The prevalence of cerebellar infarctions, strokes of different ages, and hemorrhagic transformation did not show marked differences between groups.

MRI patterns and vascular territory involvement according to the PFO status are compared in Table 14.

**Table 14:** MRI patterns in ESUS–PFO versus ESUS non-PFO. MRI: magnetic resonance imaging, ESUS: embolic stroke of unknown source, PFO: patent foramen ovale, MCA: middle cerebral artery, PCA: posterior cerebral artery, PICA/AICA/SCA: posterior inferior cerebellar artery/anterior inferior cerebellar artery/superior cerebellar artery

MRI characteristic	ESUS–PFO (n = 39)	ESUS non-PFO (n = 15)
Multifocal infarction, n (%)	10 (25.6%)	4 (26.7%)
Carotid territory involvement, n (%)	23 (59.0%)	10 (66.7%)
Vertebrobasilar territory involvement, n (%)	17 (43.6%)	6 (40.0%)
MCA involvement, n (%)	23 (59.0%)	10 (66.7%)
PCA involvement, n (%)	10 (25.6%)	4 (26.7%)
Artery of Percheron infarction, n (%)	3 (7.7%)	1 (6.7%)
Cerebellar territories (PICA/AICA/SCA), n (%)	11 (28.2%)	4 (26.7%)
Infarcts of different ages, n (%)	9 (23.1%)	4 (26.7%)
Hemorrhagic transformation, n (%)	4 (10.3%)	1 (6.7%)

Given the small size of the ESUS without PFO group, formal statistical testing was not performed, and the results are presented descriptively.

## Discussion

In this retrospective ESUS cohort, we emphasize the value of the TCD bubble test as a sensitive and clinically useful screening tool for PFO, providing crucial diagnostic guidance and influencing subsequent management strategies. Our results support the idea that, in ESUS populations, PFO is a common and potentially significant embolic source that requires both functional and anatomical assessment for detection and characterization [7,8,9].

The prevalence of PFO observed in our cohort is consistent with previous ESUS studies, which report higher rates than in the general population, where PFO occurs in about a quarter of adults [10,11]. This overrepresentation supports the idea that PFO may contribute to stroke development in certain ESUS patients. However, growing evidence suggests that the clinical importance of PFO depends not only on its presence but also on the presence of functional shunting, high-risk anatomical features, and prothrombotic or triggering conditions [12].

From a pathophysiological perspective, PFO facilitates paradoxical embolism by allowing venous thrombi or microemboli to bypass pulmonary filtration during transient right-to-left pressure gradients [13]. Situations such as Valsalva maneuvers, prolonged immobilization, or increased pulmonary pressures can promote shunt opening and embolic passage [14]. In addition, several clinical factors commonly seen in ESUS populations may enhance this mechanism. Migraine, especially migraine with aura, has been repeatedly linked to PFO, possibly through microembolization or impaired pulmonary clearance of vasoactive substances [15,16]. The use of oral contraceptives and tobacco smoking contributes to a prothrombotic environment and endothelial dysfunction, potentially increasing the likelihood that a functionally significant shunt becomes clinically relevant [17,18]. These observations support a multifactorial model in which PFO acts as a permissive conduit, with its pathogenicity determined by associated risk factors rather than by its status as an isolated anatomical anomaly.

Within this framework, accurate detection of a right-to-left shunt is crucial. While TEE remains the standard method for assessing PFO anatomy, its invasive nature, need for sedation, and common difficulty in performing an effective Valsalva maneuver can limit its sensitivity in routine clinical practice [19]. By contrast, TCD with the bubble test offers a non-invasive, bedside, and highly sensitive way to detect functional shunting, with excellent patient comfort. Several studies have demonstrated that TCD's sensitivity is comparable to, or even better than, that of TEE in identifying right-to-left shunts, especially for small or intermittent shunts [20,21].

Our results align with these observations and further emphasize the complementary roles of TCD and TEE. The discrepancies observed between TCD and initial TEE in our cohort highlight the limitations of relying on a single diagnostic method. Importantly, repeating TEE in selected discordant cases improved diagnostic agreement, suggesting that a sequential diagnostic approach-systematic TCD screening followed by targeted TEE and reassessment when needed-may enhance PFO detection [22]. This strategy allows TCD to serve as an effective gatekeeper, identifying patients who are most likely to benefit from invasive anatomical evaluation while reducing unnecessary procedures. In addition, in one patient with a severe right-to-left shunt detected by TCD despite a negative TEE, further evaluation with chest computed tomography revealed a pulmonary arteriovenous malformation consistent with hereditary hemorrhagic telangiectasia, demonstrating that TCD detects functional right-to-left shunt regardless of its anatomical source [22].

Our data suggest that TEE might more often miss small right-to-left shunts. The predominance of low-grade shunts in our discordant cases implies that the size of the shunt and the effectiveness of the Valsalva maneuver may affect the accuracy of echocardiographic detection [22,23].

An additional finding of our study relates to patient cooperation during the Valsalva maneuver. We observed better Valsalva performance during TCD examinations than during TEE, probably because there was no sedation and patients felt more comfortable during TCD. This difference may explain the conflicting results seen between TCD and initial TEE in some patients. Suboptimal Valsalva during TEE has been previously identified as a significant limitation, potentially decreasing the sensitivity of echocardiography in detecting a right-to-left shunt [24]. In contrast, TCD allows real-time monitoring of changes in cerebral blood flow velocity, providing objective confirmation of an effective Valsalva maneuver and enhancing functional shunt detection [25].

Beyond diagnosis, using TCD has direct implications for treatment decisions. Classifying shunt severity and identifying high-risk patterns, such as severe shunt or curtain effect, helps evaluate risk and select patients for more intensive secondary prevention. In our group, this approach guided multidisciplinary decisions regarding anticoagulation and percutaneous PFO closure, aligning with current recommendations that emphasize personalized management based on combined clinical, functional, and anatomical factors [25-27].

Notably, a small but significant proportion of patients undergoing percutaneous PFO closure in our study were aged 60 years or older. The closure was considered only when high-risk anatomical and/or functional features were present and after multidisciplinary discussion [28]. Although PFO prevalence decreases with age in the general population, emerging data suggest that PFOs that persist into older age are more often linked with high-risk features and have greater embolic potential. This is supported by a post-hoc analysis of the DEFENSE-PFO trial, which showed a benefit of closure even in patients aged ≥60 years if high-risk PFO characteristics were present [29]. While these findings remain exploratory, they encourage a shift from strict age thresholds toward a pathophysiology-driven approach in which TCD-derived functional assessment plays a key role [30,31].

Several limitations need to be acknowledged. The retrospective design and lack of blinded imaging interpretation could introduce bias, and subgroup analyses were limited by sample size. Imaging interpretation was not blinded to clinical or Doppler findings, which may have introduced observer bias. However, this reflects real-world clinical practice. Nonetheless, the standardized TCD protocol, systematic evaluation of discordant cases, and comprehensive diagnostic work-up enhance the reliability of our findings.

Overall, our findings highlight the crucial role of TCD with the bubble test in diagnosing ESUS. By providing a sensitive, non-invasive way to assess right-to-left shunt, TCD greatly improves PFO detection, guides TEE use, and supports personalized treatment plans. These results recommend using TCD as a primary screening tool for ESUS and call for more prospective studies to refine TCD-based diagnosis and management protocols.

In addition, data on PFO detection and right-to-left shunt assessment in ESUS populations from African centers remain scarce. Our study contributes real-world evidence from a North African tertiary center, supporting the applicability and diagnostic value of TCD in diverse healthcare settings.

## Conclusions

In patients with ESUS, the TCD bubble test serves as a highly sensitive, non-invasive screening tool for detecting right-to-left shunt and plays a crucial role in diagnosing PFO. By providing a dependable functional assessment, TCD complements anatomical imaging, clarifies discordant echocardiographic results, and guides appropriate use of TEE. Our findings support the adoption of TCD as a primary screening method in ESUS diagnostic pathways and emphasize its clinical importance for patient stratification and management, including the selection of candidates for percutaneous PFO closure. Additional prospective studies are needed to validate TCD-guided diagnostic algorithms and refine personalized treatment strategies across various age groups.
